# Comprehensive and quantitative analysis of G_1_ cyclins. A tool for studying the cell cycle

**DOI:** 10.1371/journal.pone.0218531

**Published:** 2019-06-25

**Authors:** Elisabet Bállega, Reyes Carballar, Bàrbara Samper, Natalia Ricco, Mariana P. Ribeiro, Samuel Bru, Javier Jiménez, Josep Clotet

**Affiliations:** Basic Sciences Department, Faculty of Medicine and Health Sciences, Universitat Internacional de Catalunya, Barcelona, Spain; Texas A&M University College Station, UNITED STATES

## Abstract

In eukaryotes, the cell cycle is driven by the actions of several cyclin dependent kinases (CDKs) and an array of regulatory proteins called cyclins, due to the cyclical expression patterns of the latter. In yeast, the accepted pattern of cyclin waves is based on qualitative studies performed by different laboratories using different strain backgrounds, different growing conditions and media, and different kinds of genetic manipulation. Additionally, only the subset of cyclins regulating Cdc28 was included, while the Pho85 cyclins were excluded. We describe a comprehensive, quantitative and accurate blueprint of G_1_ cyclins in the yeast *Saccharomyces cerevisiae* that, in addition to validating previous conclusions, yields new findings and establishes an accurate G_1_ cyclin blueprint. For the purposes of this research, we produced a collection of strains with all G_1_ cyclins identically tagged using the same and most respectful procedure possible. We report the contribution of each G_1_ cyclin for a broad array of growing and stress conditions, describe an unknown role for Pcl2 in heat-stress conditions and demonstrate the importance of maintaining the 3’UTR sequence of cyclins untouched during the tagging process.

## Introduction

The cell cycle–the basis for perpetuating life–can be described as a set of events unfolding in a cell that result in the generation of two new cells that are virtually identical to the original. Cell cycle processes are tightly controlled to produce viable cells as nearly identical as possible to the original. Cell cycle control is mastered by a family of proteins called cyclin dependent kinases (CDKs), responsible for the phosphorylation of many different substrates directly involved in processes that successfully multiply cells [1–5]. Despite their structural similarity, not all CDKs are involved in cell cycle progression [6].

The main focus of this work is not CDKs as such but cyclins, a family of proteins capable of making specific complexes with the CDKs and in charge of controlling their activity [7–11]. Cyclins are so called because of their oscillatory presence in the cell cycle, due to specific sequences and regulators in both their promoters and the three prime untranslated regions (3’UTR) (for reviews see [12, 13]) and to post-translational regulation [14]. The diversity and oscillation of cyclins are thought to be crucial for substrate selection by the CDKs and, consequently, in triggering the orderly succession of events that is essential for faithful cell cycle progression and cell multiplication [15]. Nevertheless, increasingly available experimental data indicate a more complex scenario in the orderly regulation of cell cycle events than mere succession in cyclin expression. A quantitative model has been proposed based on increasing CDK activity along the cell cycle and using different thresholds in the affinity of the different cell cycle phase substrates to be phosphorylated [16–18]. Intermediate kinase/phosphatase ratio models have also been described based on balancing CDK phosphorylation activity and counteracting phosphatase activity [19].

Cyclins are grouped into families according to different criteria. Based on temporal expression and action, we can distinguish between G_1_ cyclins [20, 21], S-phase cyclins [22, 23], G_2_ cyclins and M-phase cyclins [24]. Specific cyclin families modulate the activity of Cdc28, the main CDK in *S*. *cerevisiae* [21, 25], and other cyclin families are specifically related to the other CDK involved in cell cycle progression in the G_1_ phase in budding yeast, namely Pho85 [15, 26]. Cdc28 is modulated by two families of cyclins that are also structurally different, namely, Cln1-Cln3 and Clb1-Clb6, while Pho85 is regulated by Pcl1-Pcl10 and Pho80. Cdc28 cyclins bear two conserved sequences called the ‘cyclin box’, responsible for cyclin-CDK physical interaction; in contrast, Pho85 cyclins have only one conserved sequence [26, 27]. Temporal expression and correlation between expression and cell cycle phases for Cdc28 and Pho85 has been documented to be as follows: in G_1_, Cdc28 is modulated by Cln1, Cln2 and Cln3, whereas Pho85 is activated by Pcl1 and Pcl2; in the S-phase, Cdc28 is modulated by Clb5 and Clb6 and Pho85 is (probably) activated by Pcl7; in G_2_, Cdc28 is modulated by Clb3 and Clb4; and finally, in the M-phase, Cdc28 is controlled by Clb2 and Clb1 while Pho85 is (apparently) controlled by Pcl9 [19, 28, 29].

The complex scenario of several CDKs and cyclins governing the cell cycle is even more intricate in the case of mammalian cells [30–32]. A long-standing question is why so many CDK/cyclin complexes are needed at a particular moment of the cell cycle; in other words, are they redundant or are they specific? [28, 29].

Regarding cyclin expression, limited quantitative information is available, although it has been established that Cln3 expression is low compared to that of other Clns; in one study, a set of tagged Clns with the 3’UTR intact was produced in an attempt to understand the difference in Cln3 regulation compared to Cln1and Cln2 regulation [33]. Subsequently described was an analysis of all Cdc28 cyclins in a set of strains where the 3’UTR was deleted [34]. Overall, the general picture of cyclin demeanour comes from a patchwork of data obtained in different laboratories that have used different strain backgrounds, different growing conditions, different detection systems and different kinds of genetic manipulation. As for the Pho85 cyclins, only qualitative data are available regarding the cell cycle phase where these are expressed [26, 28]. To date, therefore, no research has comprehensively quantified and studied all the G_1_ cyclins (both Clns and Pcls) together.

In an attempt to inject some coherence into the data on the *S*. *cerevisiae* G_1_ cyclins, we designed and produced a set of strains and identically tagged the different cyclins in order to comparatively analyse their levels of expression. We selected a clean tagging system, respectful of the 3'UTR sequence and designed to avoid some of the artefacts detected in an earlier phase of our research (these artefacts, which are not considered in the current G_1_ cyclin expression model, are discussed further below). We used our set of strains to shed some light on the question of the specificity or redundancy of cyclins and CDKs in different environmental conditions and identified a previously unreported role for Pcl2 in cell cycle progression at high temperatures. Below we describe a tool, in the form of a set of identically tagged strains, for studying the G_1_ phase and how cell cycle progression may be affected by environmental stress conditions or external treatments such as chemotherapeutic drugs.

## Results

### G_1_ cyclin tagging

In our endeavour to provide the cell cycle community with a unified and comprehensive study of the pattern of expression of all G_1_ cyclins in *S*. *cerevisiae*, we investigated the G_1_ cyclins in exactly the same growing conditions, using exactly the same tagging strategy and using a single and widely used *S*. *cerevisiae* genetic background, namely, BY4741.

**Fig 1:**
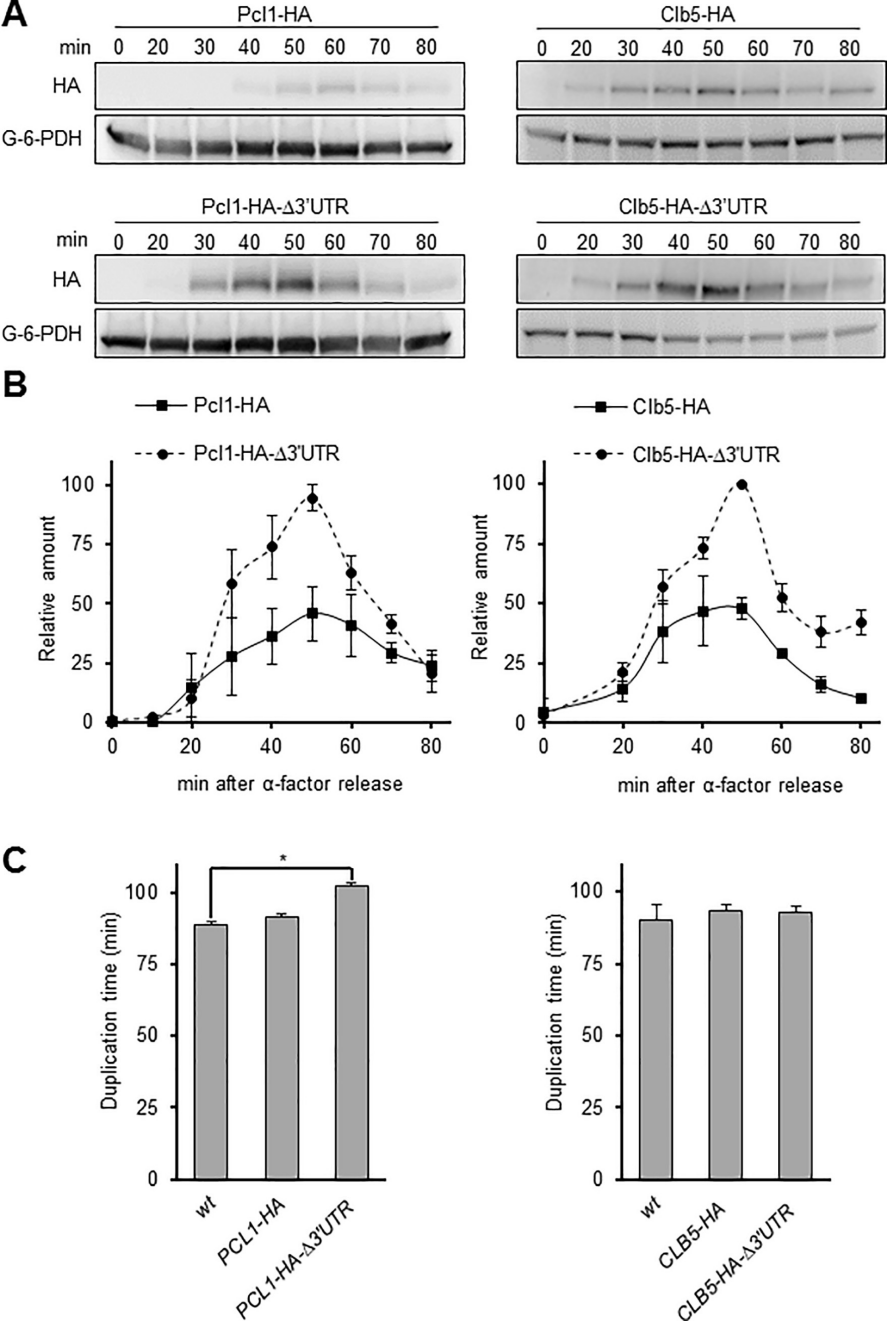
The 3’UTR sequence of cyclin genes is important for protein levels. A) Protein amounts for Clb5 (right) and Pcl1 (left), Cdc28 and Pho85 cyclins, respectively. The genes of both cyclins were modified to introduce the 3HA tag sequence, either eliminating or retaining the 3’UTR sequence. Cells were grown to the exponential phase in YPD (see Materials and Methods), synchronized in G_1_ with α-factor and released in fresh YPD medium. At specific times, samples were taken and protein amounts were determined by western blot analysis using Image Studio Lite software. Representative western blot images are depicted. Although the blots for cyclins with and without 3’UTR are presented as separate images, they were realized, analysed and developed in the same membrane. B) Quantification of the amount of Pcl1-3HA and Clb5-3HA with and without 3’UTR. As in A), the signal from the western blots was quantified using Image Studio Lite software. Values were standardized using loading control and were relativized to the maximum expression amounts. Mean±SEM values for three and four independent experiments for Clb5 and Pcl1, respectively, are shown. C) Duplication time of strains bearing *PCL1-3HA* or *CLB5-3HA* with or without the 3’UTR sequence. Cells were grown overnight in YPD at 30°C, diluted to OD = 0.4 in fresh medium and incubated in a water shaker at 30°C. Samples were taken every 10 min over 420 min. Optical density (wavelength 660 nm) was used as a measure of cell density. Mean±SEM values for three independent experiments are shown. An asterisk indicates a statistically significant difference (p≤0.05).

Cyclin amounts have frequently been evaluated by tagging the cyclins at their C-terminus and inserting a marker for the selection of transformants; this separates the 3’UTR from the gene and eliminates its putative regulatory function. However, growing evidence points to the importance of the 3’UTR for gene expression and protein amount regulation in both mammalian cells [35, 36] and yeast [37] (for a recent review see [38]). Therefore, before we tagged all the cyclins, we evaluated the influence of the 3’UTR sequence on two different G_1_ cyclins. We did so by monitoring the amount and timing of Pcl1 expression in a *PCL1-3HA* tagged strain where the 3’UTR sequence was replaced by a selection marker (*KanMX*) as an inherent feature of the tagging strategy (as done in the classical strategy). We then compared this to a *PCL1-3HA* strain in which the 3’UTR was not altered using a strategy called *delitto perfetto* [39]. *Delitto perfetto* permits a DNA sequence to be tampered with while leaving no trace other than the desired modification (see Materials and Methods). Fig 1A and Fig 1B show that the amount of Pcl1-3HA was notably higher in the classically tagged strain. Similar results were obtained when the same approach was applied to *CLB5*, indicating that integrity of the 3’UTR might be key to cell regulation of the amount of cyclins and may, consequently, be a requirement for correct evaluation of the G_1_ cyclin blueprint.

To reduce interference with the amount of the protein as much as possible, we performed an analysis aimed at determining the most appropriate tag to use. TAP (a large tag) and 3HA (a small tag) detected the cyclins reasonably well. Although our results indicated that tag size does have a minor impact on the amount of Clb5, applying Occam’s razor principle for simplicity sake, we nevertheless decided to use the smaller 3HA tag. In the course of our research, it has been reported [40] that the 3HA module developed by Longtine et al. [41] uses a linker between the protein to be tagged and the 3HA epitope that greatly affects the stability of the tagged protein. In our research we used the tool-box system [42], which uses a different linker that does not affect protein stability [40].

Altering the level of cyclins is usually detrimental to normal cell cycle progression [43]. To check whether a variation in the amount of cyclins as determined by the presence or absence of the 3’UTR had any physiological impact, we studied the duplication time of cells bearing cyclins with or without the 3’UTR. As can be appreciated in Fig 1C, eliminating the *PCL1* 3’UTR produced a statistically significant increase in duplication time (from 89 to 102 min), which confirms that the extra amount of Pcl1 alters cell physiology. Interestingly, an increase in the amount of Clb5 in the strain *CLB5-3HA*-Δ3'UTR did not produce any significant difference in duplication time (in standard growing conditions at least); this is consistent with the existence of differential compensatory mechanisms depending on which cyclins are affected.

In view of the above results, we tagged each G_1_ cyclin in a separate strain (since tagging several cyclins in the same strain could have resulted in the additive effect of small perturbations) in the most respectful way possible, i.e., maintaining their 3’UTR intact and using the 3HA tag from the tool-box module. We also tagged Clb5 and Sic1 using the same strategy and used them as landmark cairns to assess the biochemical border for G_1_-S transition.

### Monitoring seven G_1_ cyclins

To reliably and efficiently monitor the seven selected proteins, we pooled the tagged strains in groups (see below). To do this, we first checked whether all the strains grew at the same rate, by growing them simultaneously in a microtiter plate at 30°C under agitation in a spectrophotometer, which permitted us to continuously monitor optical density. No statistically significant differences were detected in their duplication times (S1A Fig). Furthermore, to better validate the pooling strategy, under a microscope, we counted the cells of all the separately growing strains at different moments of the experiment and also counted the proportion of budding at 35 min after α-factor release. In all cases all the separately growing strains behaved very similarly (S1B and S1C Fig). Finally, using western blot we monitored all cyclins from the separately growing strains, finding that the pattern of expression was very similar to the pattern observed when the strains were pooled (see below and see S1D Fig).

**Fig 2:**
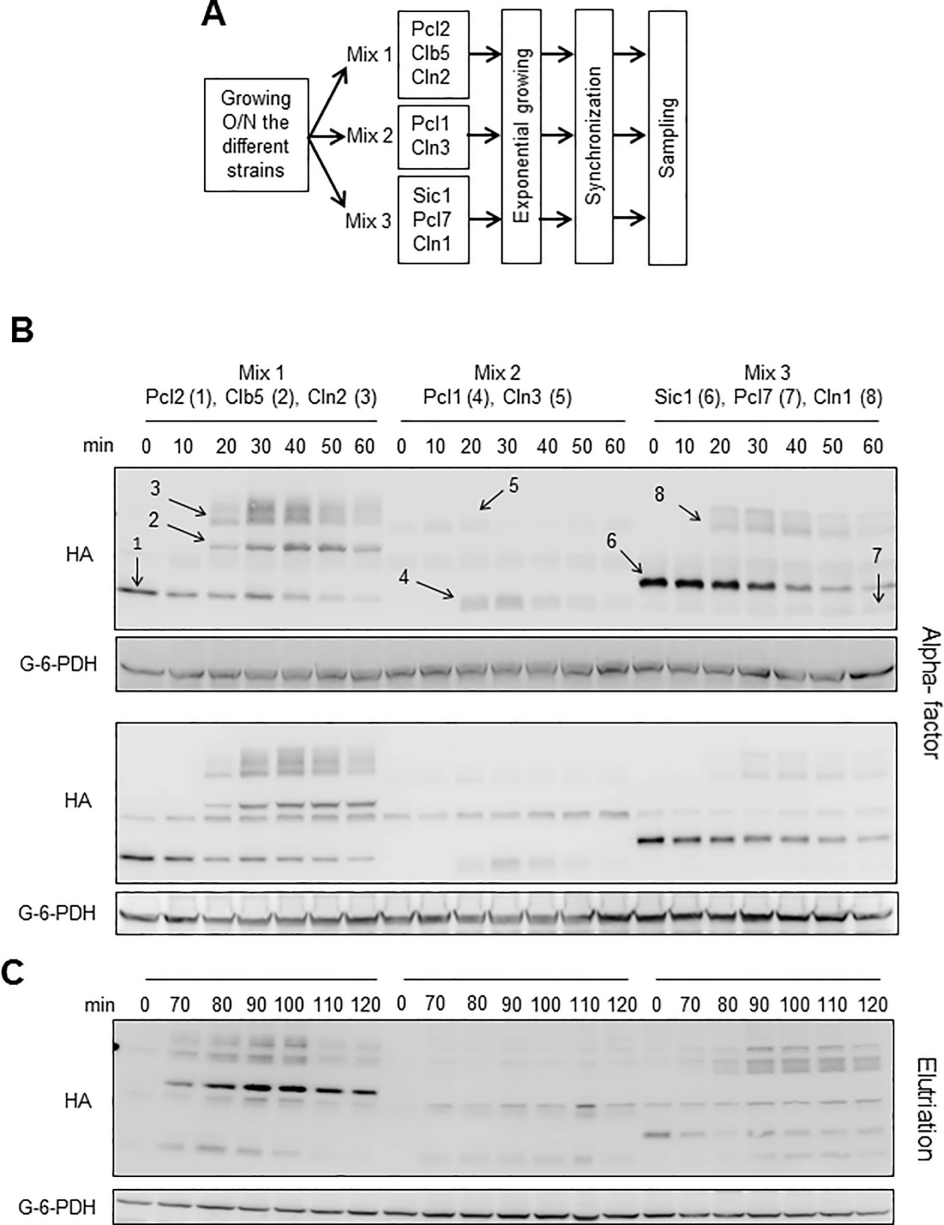
Experimental setup for determining G_1_ cyclin amounts. A) Workflow and pooling scheme. Strains were grown overnight in YPD at 30°C in a water shaker, diluted in fresh medium, mixed in three different sets according to the molecular weight of the tagged proteins, grown in the exponential phase, synchronized, released in fresh medium and subjected to the designed stress or treatment (see Materials and Methods). B) Two representative and independent western blot images (to show reproducibility) used to quantify G_1_ cyclin amounts. Cells were synchronized using α-factor and samples were obtained as described in A), separated by SDS-PAGE and blotted and developed (see Materials and Methods). The different cyclins are indicated by numbers in the upper image. C) Same procedure as in B), except that cells were synchronized by elutriation, with time 0 corresponding to the moment the cells were retrieved from the elutriation device, after which the cells were incubated under agitation at 30°C.

Overnight cultures of cells with the different tagged cyclins were pooled in strictly controlled quantities in three mixes, designed according to the size of the cyclins to ensure correct detection of all cyclins in the same blot (see Fig 2A). The mixes were grown exponentially for 3 h, synchronized in G_1_ by incubation with α-factor and released synchronously into the cell cycle. To minimize experimental noise, expression of all G_1_ cyclins was monitored in a single western blot (see representative images in Fig 2B). The specificity of signals was checked using a non-tagged strain. Note that, for the two western blot images from independent experiments included in Fig 2A, we obtained reproducible data on the relative amounts and the appearance-disappearance dynamics of the different G_1_ cyclins. This information can be considered accurate and reliable, as the cells were grown at the same time, in the same incubator, using the same polyacrylamide gel for the electrophoresis, using the same transfer conditions and the same transfer device and using the same antibody solution, developed and exposed in an identical way. Since α-factor synchronization produces intrinsic artefacts, we repeated the analysis using elutriation as a very different synchronization method, with the results depicted in Fig 2C.

In our analysis, we also included the cyclins specific for the Pho85 CDK (Pcl1, Pcl2 and Pcl7). This meant that we could obtain evidence on the relative importance of the Cln and Pcl sets of cyclins, so as to hypothesize regarding the contribution of their respective CDKs (Cdc28 and Pho85) to G_1_ progression in both normal growing conditions and, more importantly, in environmentally different growing conditions (explained further below).

### A cyclin map for G_1_/S-phase transition in *S*. *cerevisiae*

**Fig 3:**
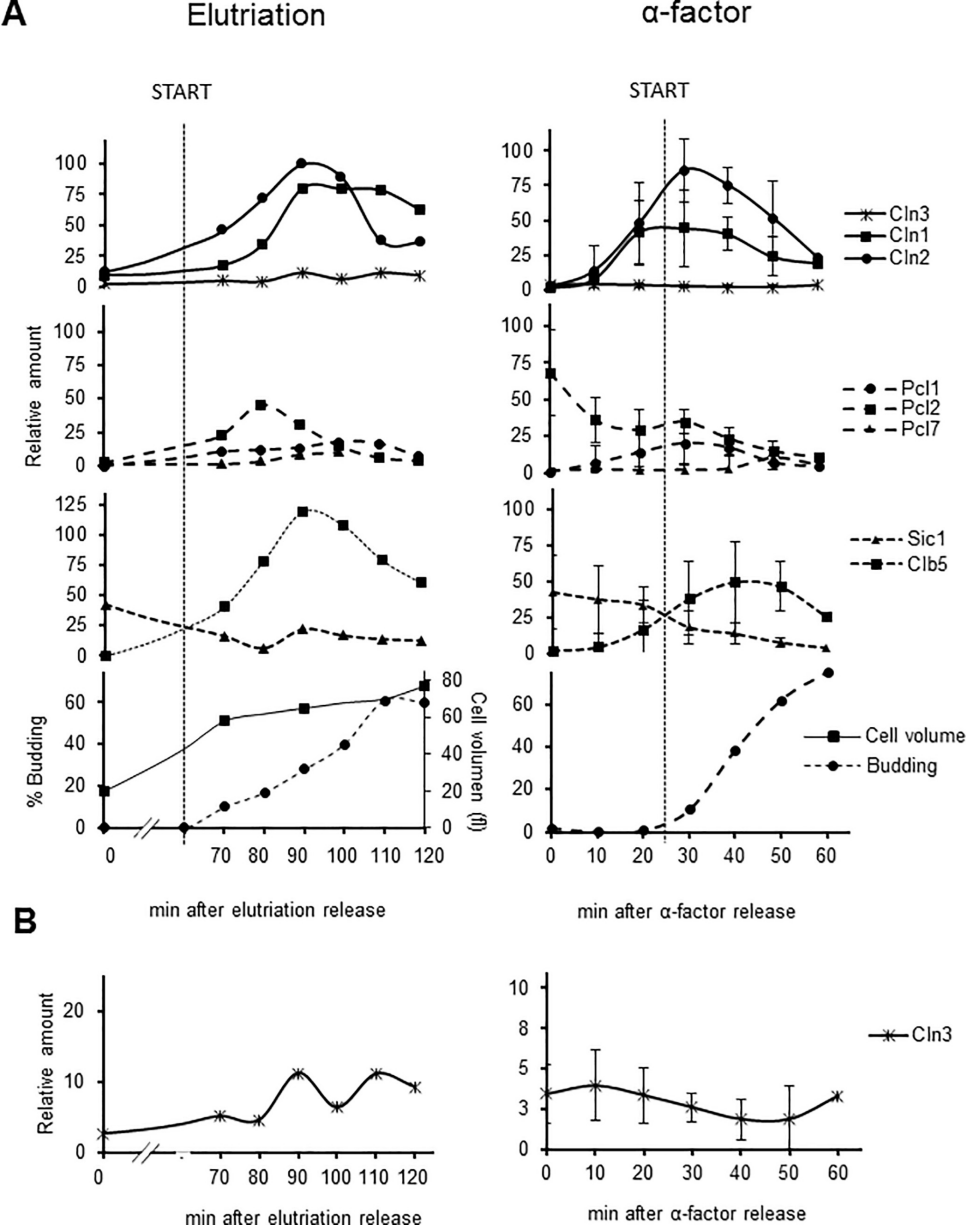
Cyclin waves in *S*. *cerevisiae* growing in normal lab conditions (YPD at 30°C). G_1_ cyclin waves as determined in this research. From top to bottom, the panels show the Cdc28 G_1_ cyclins, the Pho85 G_1_ cyclins and the different molecular markers (Clb5 and Sic1) and morphological markers (budding percentage and size for elutriation). START, determined as the moment in which Sic1 and Clb5 amounts were identical, is extrapolated as a dashed line to all the panels. A) Cells were synchronized by centrifugal elutriation (left panels) or α-factor (right panels). Time 0 corresponds to the moment of cell removal from the elutriation device or α-factor removal, after which cells were incubated under agitation at 30°C. Note that the time elapsed before the cells resumed the cell cycle was greater after elutriation than after α-factor treatment. B) Since the amount of Cln3-3HA is very low, it is plotted both with the other Clns and individually so as to clearly represent levels. In the case of α-factor synchronization, at least three independent western blot experiments as in Fig 2B were quantified, standardized using loading control and relativized to the maximum expression amounts (Cln2). Values are expresed as mean±SEM.

We quantified at least three independent experiments (in the case of α-factor synchronization)–like those depicted in Fig 2B–to produce cyclin blueprints that included both amplitude (temporal expression) and height (protein expression) of the G_1_ cyclin waves. To simplify visualization, the Cdc28 and Pho85 cyclins (Clns and Pcls, respectively) are shown in different graphs. We also used Clb5 and Sic1 levels together with the budding index (virtually identical for all the strains; (see S1C Fig) to establish the precise moment of START, defined as the moment in which Clb5 and Sic1 amounts are identical (see Materials and Methods and Fig 3). All together, these produced an accurate picture of the G_1_ cyclin universe in a yeast cell (Fig 3).

We found some differences between the two synchronization methods, specifically, the presence of Pcl2 with α-factor synchronization, as previously reported [26], a slightly delay in the expression of Cln1 compared to Cln2 and an increased level of Clb5 in elutriation. We also found some similarities: the low level of Cln3 (although higher for elutriation than for α-factor) and the bulk of Cln1 and 2 expression taking place after START. At this point, we decided to perform the rest of our experiments using α-factor synchronization method rather than centrifugal elutriation, which, although it has been used to study cyclin expression on several occasions [44, 45], is a more complex method for the purposes of the research described in this article.

Looking at the cyclin pattern resulting from α-factor synchronization, we can first confirm very low-level and slightly cyclic Cln3 behaviour [33]. Second, Cln1 and Cln2, which are typically plotted as a single curve (for simplification sake), are present in different amounts, as already reported for expression from plasmids [46] and for interference with 3’UTR sequences [47]. Third, our temporal map of Cln expression in relation to START is noticeably different to the current widely used model, with the maximum level reached in the S-phase (Fig 3A). Finally, for the first time, Pcl cyclins are included in the G_1_ cyclin picture (Fig 3A).

### Cyclin and cyclin family amounts in different conditions and stresses

The amounts of specific cyclins depend on the environmental stress conditions to which cells are exposed. Some examples are Cln2 in response to osmotic shock [48], Cln1 in response to glucose [49] and Cln1, Cln2, Cln3 and Clb5 in response to heat shock [50]. However, those studies feature some of the drawbacks mentioned above, mainly, modification of the 3'UTR and failure to include all the cyclins. To obtain a more accurate description of cyclin behaviour in stress conditions, we used the strains and setup described above, synchronized the cells using α-factor and released them under four different stress conditions: heat shock (37°C), osmotic stress (0.4M NaCl), reductive stress (100 mM N-acetyl cysteine) and oxidative stress (10 μM menadione). We also tested cyclin levels when cells were growing in different mediums: SD at 30°C and malt-based medium (as a more physiological growth medium for yeast) at 30°C and at 37°C. The corresponding analyses pointed to variations in the expression pattern of the G_1_ cyclins with respect to standard lab growing conditions (i.e., YPD at 30°C). Western blots of all the conditions and stresses analysed, along with quantifications and graphical representations of all the G_1_ cyclins in the different conditions, are depicted in S2–S5 Figs.

**Fig 4:**
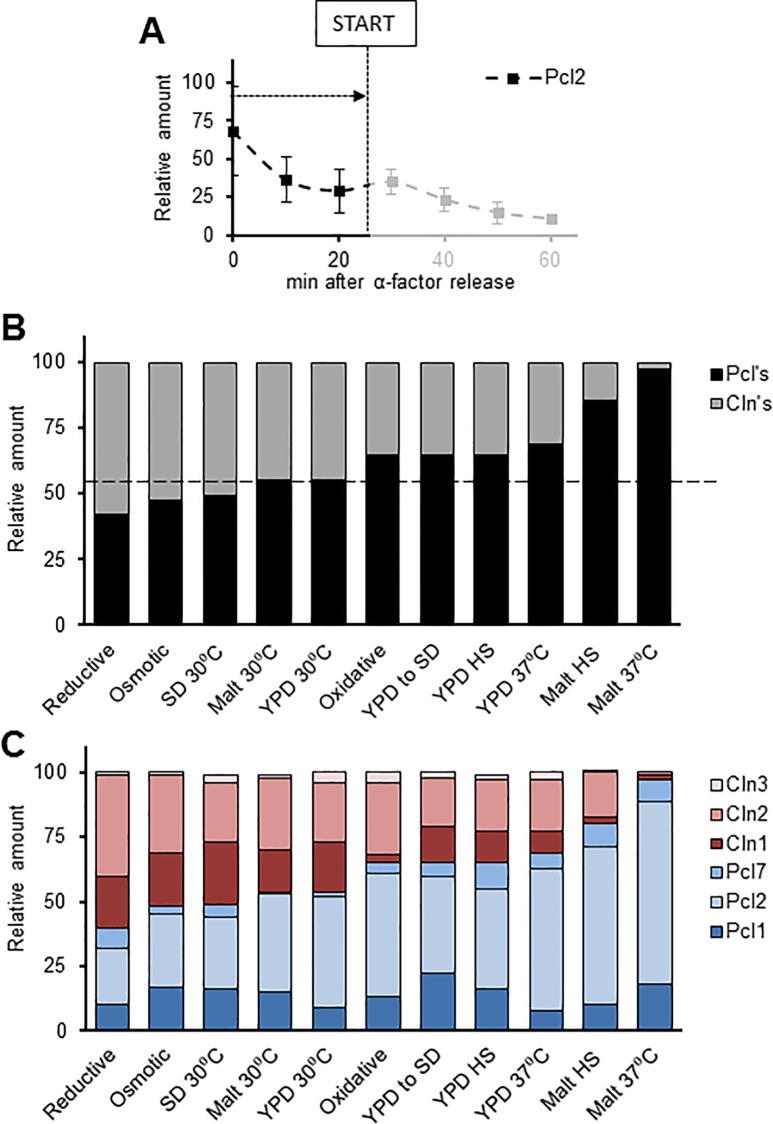
Cyclin amounts in different conditions and stresses. Strains were grown and processed as described in Fig 2B. Western blots were quantified, values were standardized using loading control, relativized to the maximum expression amounts and plotted as in Figs 1B and 3B. A) Cyclin amounts in particular environmental or stress conditions were obtained by calculating the area under the curve from α-factor release to START. B) Pcl and Cln amounts were calculated as for A). Conditions are ordered according to increasing amounts of Pcl. C) Same procedure as in A) but with the different members of the two cyclin families separated. Conditions are ordered according to increasing amounts of Pcl. Values are expressed as means of at least three independent experiments.

To produce a more comprehensive view of changes, we depicted the contribution of each G_1_ cyclin in single graphs that showed cyclin amounts from α-factor release to START (see Fig 4A for an example). In our experimental setup, Clns and Pcls were expressed at similar levels in most of the tested conditions (Fig 4B), reinforcing the idea that both groups of cyclins are necessary for correct cell cycle passage through the G_1_ phase. This scenario was generally maintained, except for heat shock and the malt-based medium: for the cells growing at a high temperature (37°C) in the malt-based medium, Pho85 cyclin amounts rose to 80% and even 90% (Fig 4B).

### Variations in cyclin family components

**Fig 5:**
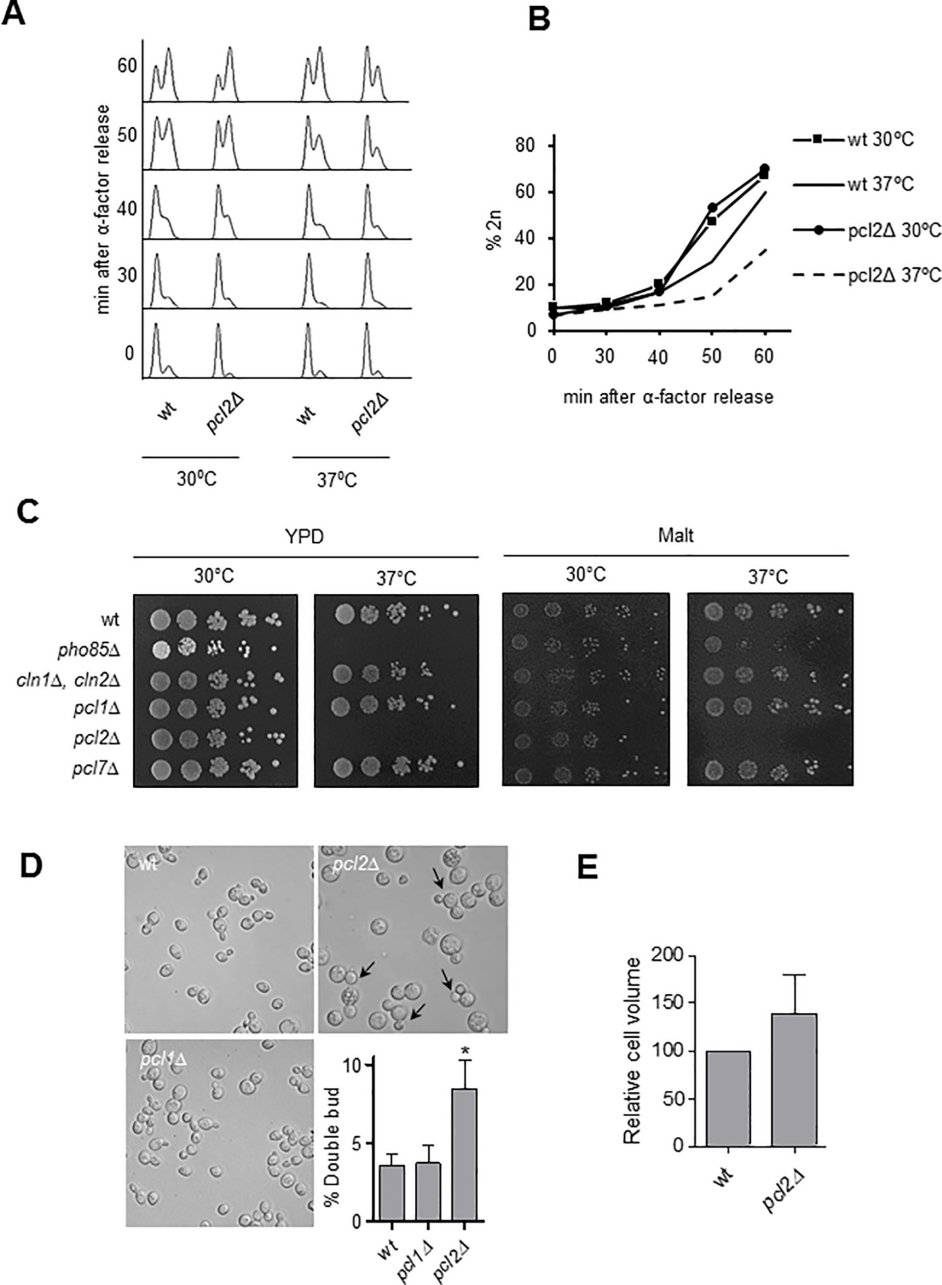
Cell cycle progression and thermosensitivity of *pcl2*Δ cells. A) FACS analysis of wild-type and *pcl2*Δ cells from the BY4741 background. Cells were grown exponentially at 30°C in YPD, synchronized in G_1_ with α-factor and released in fresh YPD medium at 30°C or 37°C. B) Quantification of cells with 2n DNA content from A). C) Spot assay. W303 background cells were grown in YPD or a malt-based medium to the exponential phase and diluted to an optical density of 0.05 (wavelength 660 nm). Spotted on plates were 5 μl of tenfold sequential dilution for incubation at the indicated temperature. D) Nomarski images of the strains after 5 h at 37°C. Arrows indicate cells showing a ‘mickey mouse’ phenotype and the bar represents 10 μm. The ‘mickey mouse’ cells were quantified (mean±SEM) for three different experiments. E) Relative cell volume (mean±SEM), based on measurement of some 30 cells from each of the three independent experiments.

Another level of complexity is represented in Fig 4C, which shows the contribution of each cyclin to the overall picture (also see Fig 4A). Noticeable is the prominent presence of Pcl2, most significantly for the malt-based medium at a high temperature. On the basis of these results we speculated that *pcl2*Δ cells might face difficulties in surpassing START when released from α-factor arrest at 37°C. The FACS analysis of cell cycle progression revealed this to be the case: *pcl2*Δ cells showed some cell cycle progression difficulties in heat-shock conditions (Fig 5A), but no difficulties in other conditions such as, for instance, osmotic stress. To evaluate the relevance of the role played by Pcl2 in high temperature conditions, we repeated the analysis using elutriation as the synchronization method (S6 Fig). Since we did not detect any notable increase in the level of Pcl2 in these conditions, we conclude that the role of Pcl2 in thermal stress is restricted to α-factor conditions.

Interestingly, the magnitude of the *pcl2*Δ cell cycle phenotype depended on the genetic background. Despite cell cycle impairment, in a spotting assay using the BY4741 background we were unable to find a growth phenotype that is clearly present in the W303 background (Fig 5C). Our results corroborate *pho85*Δ thermosensitivity, as other studies have also documented *pho85*Δ difficulties in growing at 37°C [51, 52]. Additionally, the inclusion of the different Pcl mutant strains in the dot assay growing analysis suggests that the thermosensitivity of *pho85*Δ might be determined by the regulation exerted by Pcl2, since Pcl2 deletion is the only deletion leading to thermosensitivity (Fig 5C), at least in the W303 background. Focusing on the *pcl2*Δ thermosensitive phenotype, we detected an interesting trait in the appearance of a significant proportion of *pcl2*Δ cells showing a ‘mickey mouse’ phenotype consisting of double-budded cells (Fig 5D) appearing after incubation for 5 h at 37°C. This phenotype was not present when Pcl1, another cyclin from the Pcl family, was deleted. Note that, despite the apparent increase in size of the *pcl2*Δ cells, we found no statistically significant differences when volumes were calculated using the Scepter cell counter (Millipore) or when cell diameter was directly measured under the microscope (Fig 5E).

We present the correlation between the change in Pcl2 amounts and particular physiological consequences as a proof of principle for the potential usefulness of our set of tagged strains, whether to unveil the physiological relevance of cyclins in specific environmental settings or to analyse cell cycle progression in different types of treatments (including chemotherapeutic drugs). However, Pcl2 is not the only cyclin that changes; important differences can also be appreciated in many other cyclins–e.g., Cln2 and Cln1, which clearly increase in response to reductive stress or in the SD medium (Fig 4B). These notable changes in expression will be analysed in detail in future research.

## Discussion

Several studies have been carried out to produce a view of gene expression and the proteome of yeast cells, for instance, a gene expression assay depending on the cell cycle [53]. However, despite the great value of that study, since it was designed to provide data on variations in the amount of mRNA in all genes (including G_1_ cyclins) relative to initial expression, it tells us very little about the level of expression of a particular protein relative to other proteins. For this reason, while it is useful for assessing the temporal framework of cyclin expression, it yields little information regarding relative amounts of the G_1_ cyclins.

In addition to transcriptomic analysis, efforts have been invested in analysing the proteome of yeast cells under many different conditions [54–56] and also in proteome variations throughout the cell cycle [57]. Unfortunately, however, the dynamic range of proteome approaches greatly limits the detection of low abundance proteins [58] like cyclins. Another systematic study of Cdc28 cyclins used a collection of strains in which 3’UTR was deleted [34]. However, we have not been able to find data in the literature regarding the relative amounts of the different G_1_ cyclins in *S*. *cerevisiae* throughout the cell cycle. A direct consequence is that, to date, no quantitative picture of the G_1_ cyclin waves including Pcls has been available.

### 3’UTR sequence in cyclins

Apart from the issues mentioned above, the current Cln wave model is seriously affected by technical artefacts; not only was it produced in different labs using different tags, it is also affected by the tags used and the genetic modifications inherent to the tagging procedure. The effect of using different tags (even though they had little influence in our case) has been recently pointed out for high-throughput analyses [54–56]. In the course of our research, an artefact was reported to affect the tagging of proteins [40], specifically, that a dramatic reduction occurred in the stability of tagged proteins due to the presence of a particular linker sequence in the 3HA module used for the Longtine system [41], for which it was reported that the use of a different linker sequence (as in our case) had less impact on the amount of proteins. It should be noted that a seminal study on the level of Clns [33] published by the Futcher laboratory did not use the Longtine tagging system but a genomic tagging approach [59] that also keeps 3’UTR sequences intact.

Importantly, our results demonstrate a role for 3’UTR sequences in cyclin genes in the amount of the proteins coded by them. The regulatory role of this gene sequence in stabilizing the mRNA and, consequently, in regulating protein levels [60, 61]–and also in relation to the proteins involved in the cell cycle [43]–is well known. We have shown that disrupting the 3’UTR of cyclins appears to have no effect either on expression timing or cyclin destruction, which may suggest that 3’UTR disruption affects mRNA stability more than promoter regulation or destruction mechanisms. However, although an interesting topic, it was not our aim to understand the molecular mechanisms behind cyclin level regulation by the 3’UTR. What is relevant to our research is the fact that the 3’UTR role points to an important issue in cyclin analysis. As one example, if the level of Clb5 is altered by affecting the 3’UTR, and this level, relative to the CDK inhibitor Sic1, is essential to defining S-phase entry, then relocating this landmark would appear to be necessary. Changes like this are likely to have a major impact on modelling studies.

The fact that it has been reported for a G_2_ cyclin that protein increases when the 3’UTR sequence is eliminated [54] would suggest a more general scenario rather than one confined to yeast. The deletion of the 3’UTR in cyclins D, B and CCND1 in mammalian cells leads to significant upregulation of the proteins [62–64]. The translational repression of the 3’UTR in cyclin B, among many other genes, has also been demonstrated for *Xenopus* oocytes [65], although the opposite has also been reported, namely, a role for the 3’UTR in the stabilization of cyclin E [66].

### A quantitative blueprint of G_1_ cyclins

To perform a cell cycle analysis like that performed for this research, the cells must first be synchronized. Several methods for doing this exist, each with their intrinsic strengths and weaknesses [67, 68]. Choosing a method therefore results in a limitation inherent to the experimental design. We chose α-factor synchronization–while accepting the limitation of synchronizing cells in the late G_1_ phase–for several reasons: first, α-factor arrest is a physiological situation for yeast, especially when they are growing in nature, but is not the case for most of the remaining systems (drugs, temperature sensitive mutants, elutriation, etc); second, since α-factor arrest has been commonly used for cell cycle studies, a great deal of evidence is available regarding its use, so a comparison with the existing model should be performed using the same approach; and finally, α-factor arrest is easy to perform and is highly reproducible. With the aim of generating a more general view that considers synchronization artefacts, we also investigated cyclin waves using elutriation synchronization. Some differences with the α-factor method included the higher amount of Clb5, the delayed expression of Cln1 and the already documented different behaviour of Pcl2 [26] (very elevated at the moment of α-factor release but reflecting a more typical cyclin wave pattern in elutriation). We also found some similarities in the two methods: the previously documented low level of Cln3, the shifted expression peak of Cln1 and Cln2 with respect to START and the very low levels of Pcl1 and Pcl7. In sum, our comparison of data from different synchronization methods suggest that cyclin wave studies need to be interpreted with care and should bear in mind the synchronization method.

Another important assumption on which our research is based is that we used the biochemical definition of START: the moment in which relative amounts of Clb5 and Sic1 are the same [69, 70]. Our evidence indicate that this definition is in good agreement with the budding index, a morphological parameter for assessing START [71, 72]. Note that this definition also reflects a subcellular definition of START as the exit from the nucleus of the main START repressor Whi5, which is strictly necessary for the Cln1 and Cln2 positive feedback loop and S-phase entry [73].

Taking into account the above facts (or limitations), our results nevertheless demonstrate, first, that most Cln1 and Cln2 are actually produced significantly beyond START and well into the S-phase. This is not entirely surprising, given their morphogenetic role in the polarized growth taking place over a significant period of time within the S-phase [74–76]. Second, we detected substantially differing expression for Cln1 and Cln2 than proposed elsewhere for systems where the 3’UTR was respected [33] or not [46, 47]. Bearing in mind the similarity of the SBF boxes in the promoters of both genes and, consequently, their fairly similar levels of mRNA, it is tempting to speculate that the molecular nature of their differential regulation may depend on the protein sequences, as already suggested elsewhere [14]. Finally, it is interesting to note that our analysis corroborates the very low levels of Cln3—in comparison with the rest of the cyclins—reported years ago [33, 77].

### Pcls: The overlooked cyclins

The Pho85 CDK is not considered essential in standard lab growing conditions, which may explain why its cyclins have never been represented in cyclin blueprints. Nevertheless, the fact that the presence of several families of CDKs controlling passage through G_1_ has been conserved through evolution to mammals [78] would point to the importance of these CDKs. One of our goals was to account for the corresponding knowledge gap by producing a full map of the G_1_ cyclins that included Pcls and, in this way, to throw some light on the problem of the redundancy of the different CDK/cyclin complexes [29]. Analysis of the Pcls led to the findings discussed below.

First, in the release from α-factor arrest, the relative amount of Pcls is surprisingly high, bearing in mind that Pcls are regulators of a ‘non-essential’ CDK. In practically all tested conditions, Pcls accounted for over 50% of total cyclins (taking into account the definition of START used by us). This could be interpreted in terms of an understated role for Pho85-Pcls in the biology of yeast cells. The role of master cell cycle regulator has deservedly been attributed to Cdc28 (Cdk1 in mammals), with a secondary, redundant or supporting role attributed to Pho85 as the other CDK involved in cell cycle progression. Nevertheless, according to a basic cell economy principle, the high level of expression of the Pho85 cyclins would point to a more active role for Pho85, at least when cells are released from α-factor arrest (a situation which, as mentioned before, is absolutely physiological for yeast in a natural environment). Second, contributions in terms of amounts of Clns and Pcls are clearly dependent on environmental conditions, and interestingly, Pcls seem to be very important in general terms, since they are always noticeably present; even more importantly, Pcls clearly take the lead in high temperature or heat-shock conditions.

Does this mean, therefore, that the two CDKs are more specific than redundant? That the absence of one cannot be fully compensated for by the presence of the other? And, regarding a different layer of regulation, do subtleties in cell cycle control result from the interaction of each CDK with different cyclins?

In addition to providing an accurate blueprint of the G_1_ cyclins, therefore, we also pondered the fundamental question of why eukaryotic cells have or need more than one CDK to control cell cycle progression and also more than one cyclin to control each CDK. We propose two possible explanations. One is that cell cycle machinery may incorporate a certain degree of redundancy in order to gain robustness. The other is that the different apparently redundant elements may have specific functions–related to the broad array of eventualities that all cells must cope with during their life–that are only apparent when cells are growing in particular conditions (whether in terms of stress or specific nutrients). The results reported here support the second possibility.

### Pcl2 and thermal control

Our analysis suggests specific roles for certain cyclins depending on the environmental conditions to which cells are exposed. The most striking finding was the increase in Pcl2 during both heat shock and permanent growing at a high temperature, especially when the cells grew in a more ‘natural’ condition (in a malt-based medium). As predicted by the increased amount of Pcl2, *pcl2*Δ cells had problems progressing through START and the S-phase in the malt-based medium and at 37°C (that is, the conditions in which this cyclin is highly expressed). While this effect was only slightly evident in the BY4741 background, when the same analysis was done in the W303 background, the phenotype of absent Pcl2 was striking, not only in cell cycle progression but also in thermosensitivity terms, as revealed by the dot assay. In view of this observation, we performed the dot assay for other, less widely used genetic backgrounds, such as YPH499 [79] and 1700, derived from 1783 [80] and, again, we detected partial thermosensitivity.

While we have no clear explanation for the enhanced thermosensitivity, we venture that it may depend on differences in the relative level of expression of the cyclins in the different backgrounds. Leaving aside background differences, microscopic inspection of the *pcl2*Δ cells revealed a ‘mickey mouse’ phenotype based on double-budded cells. This phenotype has been described in the absence of the GPI-anchored wall protein Gas1 [81]. Expression of Gas1, which is essential for normal cell wall synthesis, is regulated during the G_1_ phase [82, 83]. The null mutant shows a thermosensitive phenotype and reduced viability at 37°C [84–86]. Altogether, it is possible to speculate that regulation of Gas1 in G_1_ by Pcl2 in thermal stress conditions cannot be supplanted by any other G_1_ cyclin. Finally, we detected no increment in Pcl2 when we used elutriation; nevertheless, we were able to find phenotypes in *pcl2*Δ cells for heat shock in conditions where α-factor was not present, indicating a role for Pcl2 in thermal stress independently of the synchronization method used.

We found other remarkable variations in the amount of cyclins in different stress conditions. One was the increase in Cln1 and Cln2 in response to osmotic or reductive stress. Although we attempted to determine whether those increases were reflected in the appearance of a phenotype when the cyclins were deleted, we found no differences in either dot assays or growing kinetics. This result leads us to suggest the following. First, another cyclin could take over the work of Cln1 and Cln2 in dealing with these stresses, given that Cln3 alone is able to drive cell cycle progression showing only a minor G_1_ delay [33]. Second, since the variation we detected in the tested conditions was not sufficient to produce a phenotype, a more sensitive analysis is needed to reveal the importance of Cln1 and Cln2 responses to osmotic and reductive stress. Finally, although consistent in the different replications of the experiment, the variations in Cln1 and Cln2 in the tested conditions were not reflected biologically.

### A new tool for assessing G_1_

G_1_ allows cells time to check internal and external environments and to ensure that conditions are appropriate and preparations are complete before major cellular processes are undertaken in the S- and M-phases. G_1_ is important for cells to decide their fate: to enter in quiescence, to sporulate, to wait for better nutrient conditions, to check their size, or to acquire confidence about successful transit through cell division and not perform this process blindly. In metazoans, misregulated G_1_ can lead to developmental problems and disease [87]. Yeast cells represent a good model for testing drugs or treatments affecting cell cycle progression in G_1_. Our set of strains can be used both as a tool for accurately assessing G_1_ cell cycle progression and as a testing bench for gaining biochemical insights into the mechanisms by which a compound could affect the expression patterns of cyclins and, consequently, cell cycle progression.

To sum up, we produced a set of strains tagged in the most respectful way possible so as to produce a quantitative and accurate picture of the G_1_ cyclins for a broad array of environmental conditions. We propose using this set of strains to monitor G_1_ cell cycle progression and to study the molecular mechanisms sustaining cell cycle effects, the use of drugs, treatments, compounds, stresses, etc. All the strains are fully available upon request.

## Materials and methods

### Yeast strains

Yeast background (except when otherwise mentioned) was always BY4741 [88]. A list of all the strains used in this work is provided in Table 1. For tagging, we followed a system based on *delitto perfetto* [39]. Briefly, for a protein to be tagged, we first deleted the complete open reading frame (ORF) and replaced it with a *URA3*-*KanMX4-3HA* double marker and tag. We then transformed the deleted cells using a DNA fragment containing the eliminated ORF and fused the fragment to the previously introduced tag (3HA) sequence using 40 nucleotide flanking tails so as to allow the recombination (integration) process to take place. To eliminate the possibility of different recombination events–due to the presence of the three times repeated sequence of the HA tag potentially leading to proteins tagged with different numbers of HA repetitions–we modified the *3HA* DNA sequence but maintained the amino acid sequence. The *3HA* sequence was as follows (underlined are the bases changed to ensure the desired integration): 5’TCAGCACTGAGCAGCGTAGTCTGGGACGTCATACGGATAGGATCCTGCGTAATCTGGGACGTCATACGGATAGCCCGCATAGTCAGGAACATCGTATGGGTA3’. Tagging, furthermore, was always checked by tag sequencing. Knock-ins were grown in plates containing 1mg/ml of the antimetabolite 5-fluoroorotic acid (5-FOA; Sigma) and were confirmed by replica plating in plates containing geneticine 0.4 mg/ml (Gibco). Selected were colonies able to grow in 5-FOA and not in geneticine.

**Table 1:** Yeast strains.

Name	Background	Genotype	Source
BY4741	BY4741	*MATa his3*Δ*1 leu*Δ*200 met15*Δ*0 ura3*Δ*0*	[88]
YJJ1024	W303-1a	*MATa leu2-3*,*112 trp1-1 can1-100 ura3-1 ade2-1 his3-11*,*15*	[89]
YPC502	YPH499	*MATa ura3-52 lys2-801 ade2-101 trp1-*Δ*63 his3-*Δ*200 leu2-*Δ	[79]
1700	1700	*MATa leu2-3*,*112 ura3-52 trp1-1 his4 canI*^*r*^	This study
YEB27	W303-1a	*PCL1-3HA*	This study
YEB56	W303-1a	*PCL1-3HA-KanMX4*	This study
YEB53	BY4741	*CLB5-3HA-KanMX4*	This study
YEB11	BY4741	*sic1*::*URA3-kanMX4-3HA*	This study
YEB112	BY4741	*PCL1-3HA*	This study
YEB113	BY4741	*PCL2-3HA*	This study
YEB114	BY4741	*PCL7-3HA*	This study
YEB116	BY4741	*CLN1-3HA*	This study
YEB117	BY4741	*CLN2-3HA*	This study
YEB118	BY4741	*CLN3-3HA*	This study
YEB119	BY4741	*CLB5-3HA*	This study
YEB120	BY4741	*SIC1-3HA*	This study
YEB182	BY4741	*CLB5-TAP*	This study
YEB181	BY4741	*pcl2*::*URA3*	This study
YEB189	W303-1a	*cln1*::*URA3-KanMX4 cln2*::*URA3*	This study
YEB184	W303-1a	*pcl2*::*URA3*	This study
YEB32	W303-1a	*pcl1*::*URA3-hyg*	This study
YEB30	W303-1a	*pcl7*::*URA3-KanMX4*	This study
YNR60	W303-1a	*pho85*::*KanMX4*	This study

Classical tagging to obtain the strains *PCL1*-3HA-Δ3’UTR and *CLB5*-3HA-Δ3’UTR was performed using the tool-box system [42] (a variation of a method developed previously [90, 91]), in which the original 3’UTR of the gene to be tagged is interrupted (and consequently inactivated) by the tagging (3HA in our case), the 3’UTR from *ADH1* and the selection marker.

### Growth conditions

The growing media used were yeast extract-peptone-dextrose (YPD: 1% yeast extract, 2% peptone and 2% dextrose) and complete synthetic (SD) medium (0.67% yeast nitrogen base, 0.5% NH_4_SO_4_, 2% glucose, supplemented with amino acids for auxotrophic requirements). For solid media, 2% of agar-agar (w/v) was added and melted during the autoclave process. For the malt-based medium plates, 1 g of malt medium from Bulldog Brews was dissolved in 7.69 ml of distilled water. For the 5-FOA plates, SD with all the required amino acids was supplemented with 5-FOA 1 mg/ml, sterilized by filtering, and added to the medium just before plating. Cells were always grown, except when otherwise specified, at 30°C under vigorous agitation (200 rpm) in water shakers. To reduce experimental variability, the overnight yeast cultures were always inoculated in strictly controlled conditions: the cells to be inoculated were obtained from a fresh colony and the number of cells to be inoculated was always constant (OD_660_ = 0.01).

### Cell synchrony, flow cytometry analysis and size measure

To synchronize the cells in G_1_, yeast cultures were grown exponentially in YPD or SD at a density of 1×10^7^ cells/ml, treated with α-factor (Biomedal) to a final concentration of 20 μg/ml; after 100 min, the cells were collected, washed and released into fresh medium to resume the cell cycle in a synchronous manner. Afterwards, aliquots were collected and processed as described elsewhere [92]. DNA was stained with propidium iodide and analysed in a FACS Calibur cytometer (Becton Dickinson) as described elsewhere [93].

Centrifugal elutriation was performed as described elsewhere [94], using a Beckman-Coulter J-26XPI centrifuge equipped with a JE-5.0 elutriator rotor. Briefly, one litre of cells was incubated in YPD under continuous agitation at 30°C during 16 h, to arrival at an optical density of around 6 (660 nm wavelength). The culture was brought into the elutriator using a peristaltic pump and equilibrated at 1900 rpm at 20°C. The G_1_ cells were then obtained from the elutriator by increasing the pump flow (total elutriation process time was 2 h). Synchrony was checked in situ by microscopic inspection. The different unbudded fractions were collected and mixed until the needed number of cells was obtained. Cells were diluted in YPD to an optical density of 1. Synchrony was checked by FACS analysis. Cells were immediately incubated in an agitated water bath at 30°C and aliquots were taken for western blot analysis.

The size of the elutriated cells was assessed using a Scepter Cell Counter (Millipore) and strictly following manufacturer indications. Briefly, cells to be measured were diluted in PBS buffer at a final concentration of around 10^5^ cells/ml.

### Cell extract and immunoblot

One ml of the yeast cell culture (1×10^7^ cells) was treated with 10 M trichloroacetic acid (TCA) to a final concentration of 20% (v/v) for 10 min and centrifuged at full speed for 1 min. The resulting pellets were dissolved in 100 μl of 0.5% SDS, 42 mM Tris-HCl at pH 6.8. Then 300 μl of glass beads (Sartorius, BBI-8541701) were added, bead-beaten twice at maximum force for 30 sec and boiled for 5 min. Around 40 μg to 60 μg of protein from each sample was separated at 90 V (10% polyacrylamide/SDS gel) and transferred to PVDF membranes (Immobilon-P; Millipore). The primary antibodies used were anti-HA 1:100 (12CA5), anti-PAP 1:4.000 (Sigma, P1291), anti-PSTAIRE 1:5000 (Abcam, ab10345) and anti-G6PDH 1:500 (Sigma, A9521). The secondary antibodies used were donkey anti-goat-HRP, donkey anti-mouse-HRP and goat anti-rabbit-HRP 1:25000 (all from Jackson Laboratories). Immunoblots were developed using Luminata Forte Western HRP Substrate (Millipore) and images were taken using GeneSnap (Syngene) and quantified using Image Studio Lite (Li-Cor).

### Measuring relative amounts of cyclins

To detect all cyclins in the same blot, we mixed different strains in the same test tube. Due to their different levels of expression we included different quantities as follows: in mix 1, 0.1 OD of each strain to a final cell concentration of OD_660_ = 0.3 (0.3 ODs); in mix 2, 0.1 OD and 0.2 OD from the strains bearing *PCL1-3HA* and *CLN3-3HA*, respectively; and in mix 3, 0.05 OD from *CLN1-3HA*, 0.1 OD from *PCL7-3HA* and 0.15 OD from *SIC1-3HA*. Note that we inoculated the same number of cells for the overnight culture and before mixing the strains, and we also checked that all cells had grown to the same extent to ensure that culture phase influence was minimized (see Fig 2A). The different quantities of cells included in the mixes were mathematically corrected after western blot quantification to revert any differences introduced in mixing.

The sampling and sample processing for separation by SDS-PAGE was as described above and in Fig 2A and Fig 2B. The bands were quantified using Image Studio Lite (Li-Cor), the amounts were corrected according to mixing (see immediately above), normalized according to load control, and relativized to the maximum signal in the blot. Using this data, we produced plots representing the relative amounts of cyclins in relation to time after release.

To produce the bar graphs representing the total amounts of the cyclins, we used the plots representing relative amounts over time, determined the START point (defined as the moment when Sic1 and Clb5 amounts were identical) and integrated the area under the curve up to START using GraphPad Prism 5 software. The obtained values represent the amounts of each cyclin at START.

### Growth curves and duplication time

Strains were grown in YPD medium overnight and were diluted to OD_660_ = 0.01. Culture cell density at 660 nm wavelength was measured continuously for 24 h under agitation at 30°C in a spectrophotometer (Biotek Synergy HT). Once cultures were growing in the exponential phase, data were plotted and duplication time was calculated.

### Dot assays

Strains were grown in YPD medium overnight, diluted to OD_660nm_ = 0.05 and tenfold sequentially diluted in fresh YPD. Spotted in the appropriate plates were 5 μl of culture for incubation at the desired temperature for 24 h or 48 h.

### Statistical analysis

Data were expressed as mean±standard error of the mean (mean±SEM). Statistical significance was determined using the Mann-Whitney U test. A *p* value of less than 0.05 was considered significant.

## Supporting information

S1 Fig Validation of the pooling strategy. A) The presence of the 3HA tag (obtained by *delitto perfetto*, then keeping the 3’UTR intact) does not significantly alter duplication time in any of the strains used. Cells were grown overnight in YPD at 30°C, diluted to OD = 0.1 in fresh medium and incubated at 30°C in a thermostated spectrophotometer under constant agitation. Optical density (wavelength 660nm) constantly measured for 420 min was used as a measure of cell density. Mean±SEM values for three independent experiments are shown. B) Number of cells, counted in a Newbauer chamber for four independent experiments, at indicated moments of the experiment (immediately after the O/N culture dilution, before α-factor addition for synchronization and at the moment of α-factor release). Values are expressed as mean±SEM for four independent experiments. C) Proportion of cells budding 35 min after α-factor release, reported as mean±SEM values for four independent experiments. D) Comparison of western blot signals obtained from pooled and individually growing strains. Exponentially growing cultures were synchronized and release and aliquots were taken at the indicated times. E) Quantification of D). Note that, other than the fact that the amount of protein could be affected by using different blots, there was no difference in expression time of the cyclins depending on the pooling strategy.
(PDF)

S2 Fig Cyclin amounts in different growing media. Representative western blot for the different mixes of cells growing in different culture media (YPD, SD and malt). Experiments were performed as described in Fig 2A and Fig 2B. Mean±SEM values quantify at least three independent experiments.
(PDF)

S3 Fig Cyclin amounts in different growing media in heat-shock or high temperature conditions. Same procedure as for S2 Fig. In heat-shock conditions, cells were grown at 30°C and then moved to 37°C on α-factor release. In heat-stress conditions, cells were exponentially grown at 37°C and temperature was kept constant after α-factor release. A representative western blot is depicted. The graphs show mean±SEM values for at least three independent experiments.
(PDF)

S4 Fig Cyclin amounts in different stress conditions. Same procedure as for S2 Fig. Cells were subjected to different stresses on α-factor release: osmotic stress (0.4 M NaCl), reductive stress (100 mM N-acetyl cysteine), and oxidative stress (10 μM menadione). A representative western blot is depicted. The graphs show mean±SEM values for at least three independent experiments.
(PDF)

S5 Fig Cyclin amounts after change to growing medium. Same procedure as for S2 Fig. Cells were grown in YPD and released from α-factor arrest into an SD medium. A representative western blot is depicted. The graphs show mean±SEM values quantifying at least three independent experiments.
(PDF)

S6 Fig Cyclin amount after elutriation in normal lab conditions and upon heat shock. The noted strains were grown as described in methods section. Cells were synchronized by centrifugal elutriation. Time 0 corresponds to the moment of obtaining the cells form the elutriation device. After this moment, cells were incubated under agitation at 30°C (upper panel) or 37°C (lower panel). We took aliquots at the indicated times and processed them for western blot analysis as in the rest of the α-factor experiments.
(PDF)
