# Supplementary figures and images for: Comprehensive and quantitative analysis of G1 cyclins. A tool for studying the cell cycle
Source: PLoS One. 2019 Jun 25;14(6):e0218531. doi: 10.1371/journal.pone.0218531 (PMC6592645; doi:10.1371/journal.pone.0218531)

Fig. S1

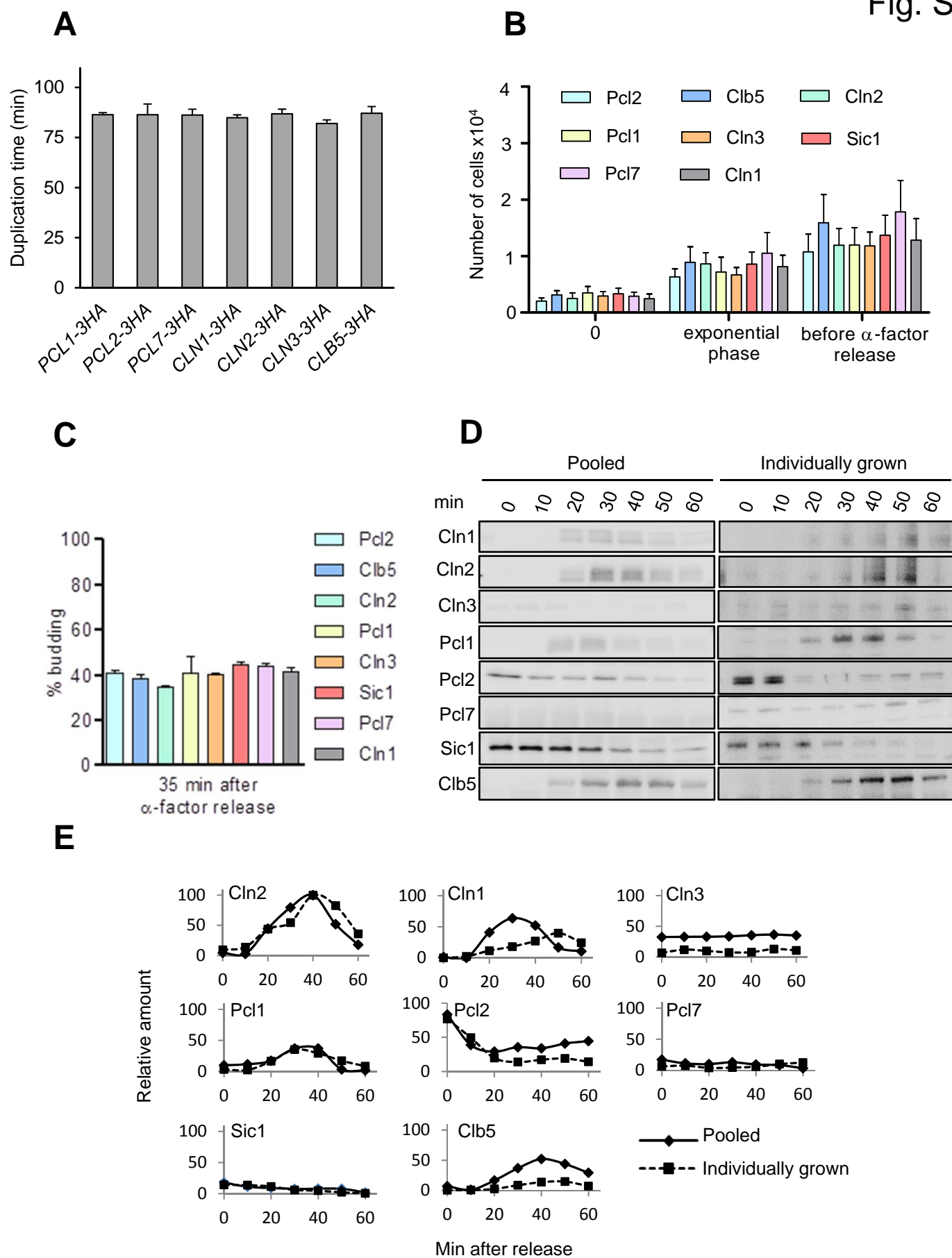

Supplement: S1 Fig — A) The presence of the 3HA tag (obtained by delitto perfetto, then keeping the 3’UTR intact) does not significantly alter duplication time in any of the strains used. Cells were grown overnight in YPD at 30°C, diluted to OD = 0.1 in fresh medium and incubated at 30°C in a thermostated spectrophotometer under constant agitation. Optical density (wavelength 660nm) constantly measured for 420 min was used as a measure of cell density. Mean±SEM values for three independent experiments are shown. B) Number of cells, counted in a Newbauer chamber for four independent experiments, at indicated moments of the experiment (immediately after the O/N culture dilution, before α-factor addition for synchronization and at the moment of α-factor release). Values are expressed as mean±SEM for four independent experiments. C) Proportion of cells budding 35 min after α-factor release, reported as mean±SEM values for four independent experiments. D) Comparison of western blot signals obtained from pooled and individually growing strains. Exponentially growing cultures were synchronized and release and aliquots were taken at the indicated times. E) Quantification of D). Note that, other than the fact that the amount of protein could be affected by using different blots, there was no difference in expression time of the cyclins depending on the pooling strategy. (PDF) [file pone.0218531.s001.pdf]

Fig. S2

**YPD 30°C**

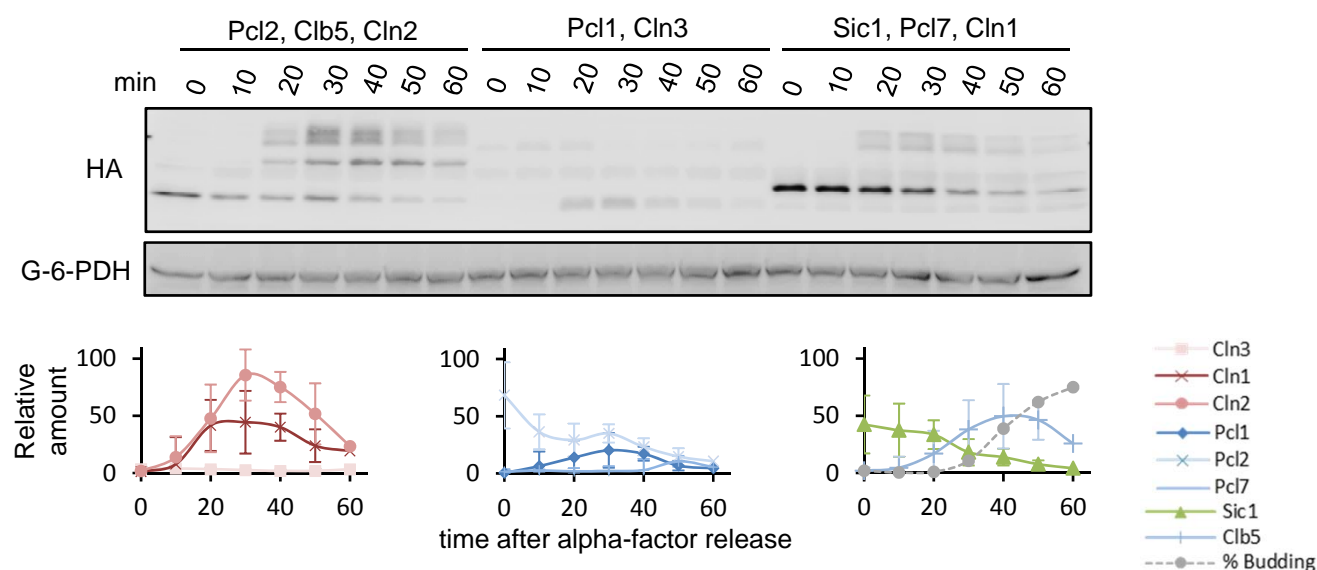

**SD 30°C**

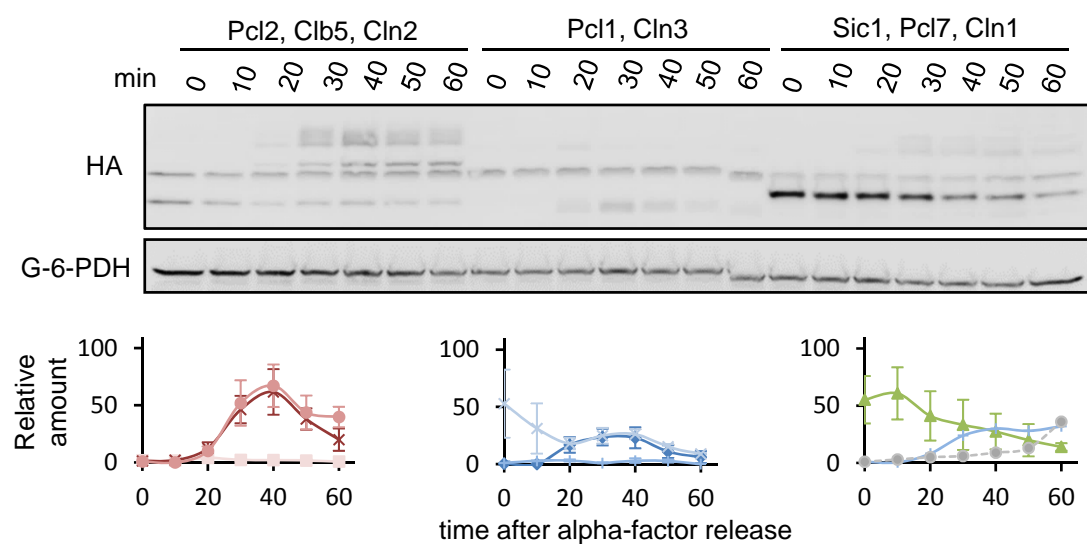

**Malt, 30°C**

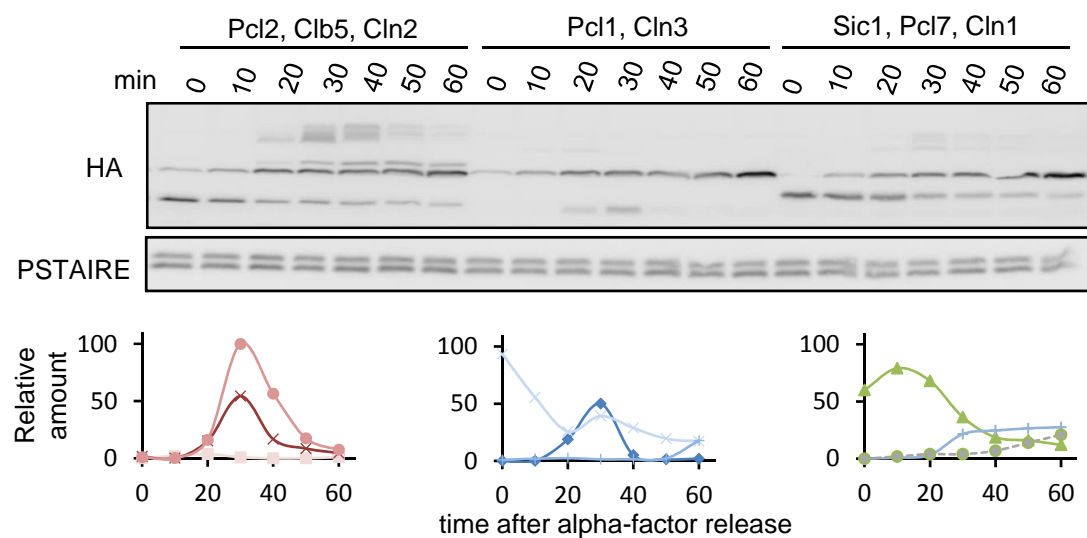

Supplement: S2 Fig — Representative western blot for the different mixes of cells growing in different culture media (YPD, SD and malt). Experiments were performed as described in Fig 2A and Fig 2B. Mean±SEM values quantify at least three independent experiments. (PDF) [file pone.0218531.s002.pdf]

**YPD 37°C**

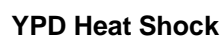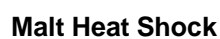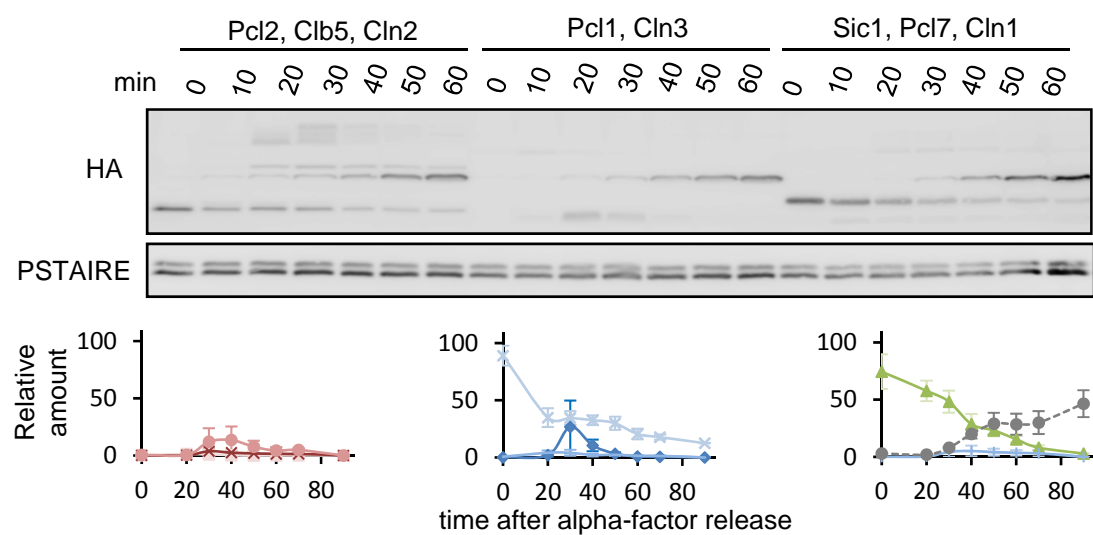

Malt, 37°C

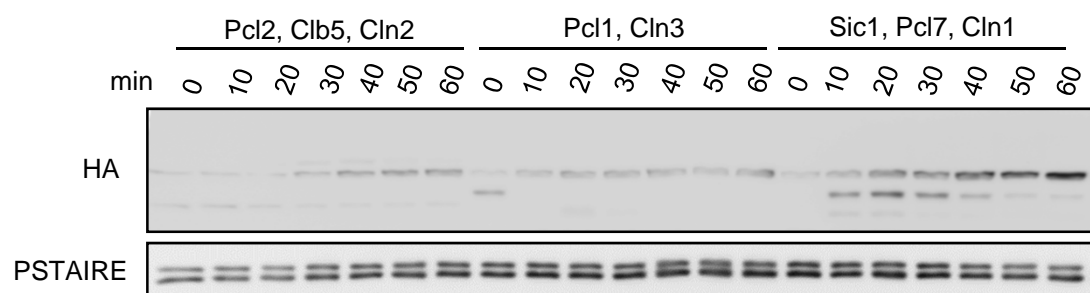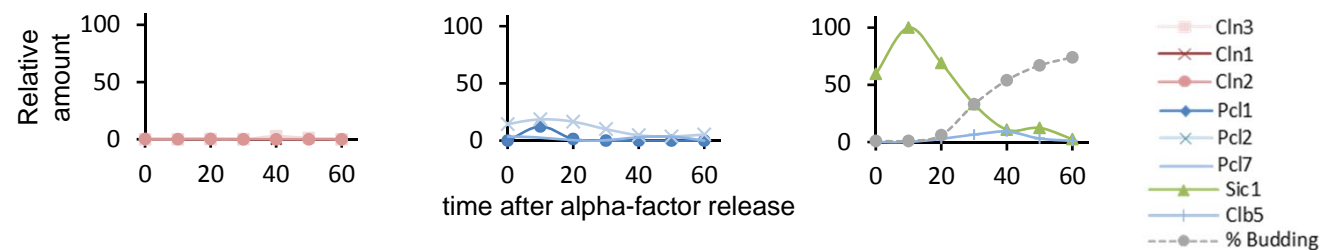

Fig. S3  
(cont.)

Supplement: S3 Fig — Same procedure as for S2 Fig. In heat-shock conditions, cells were grown at 30°C and then moved to 37°C on α-factor release. In heat-stress conditions, cells were exponentially grown at 37°C and temperature was kept constant after α-factor release. A representative western blot is depicted. The graphs show mean±SEM values for at least three independent experiments. (PDF) [file pone.0218531.s003.pdf]

Fig. S4

### Osmotic stress

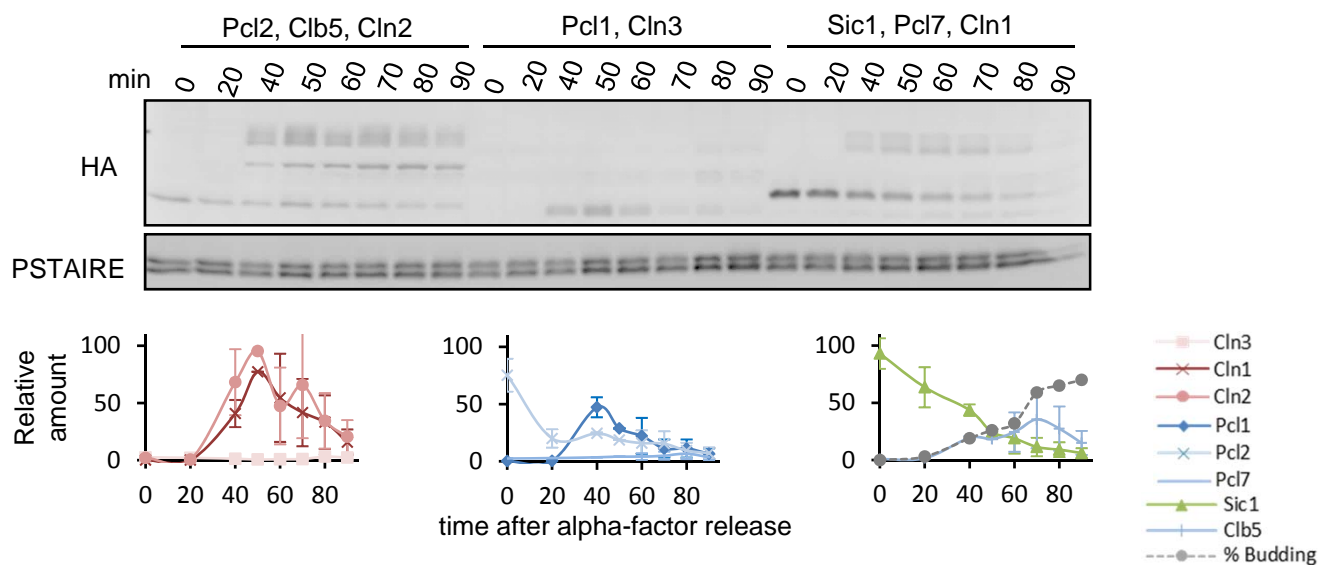

### Oxidative stress

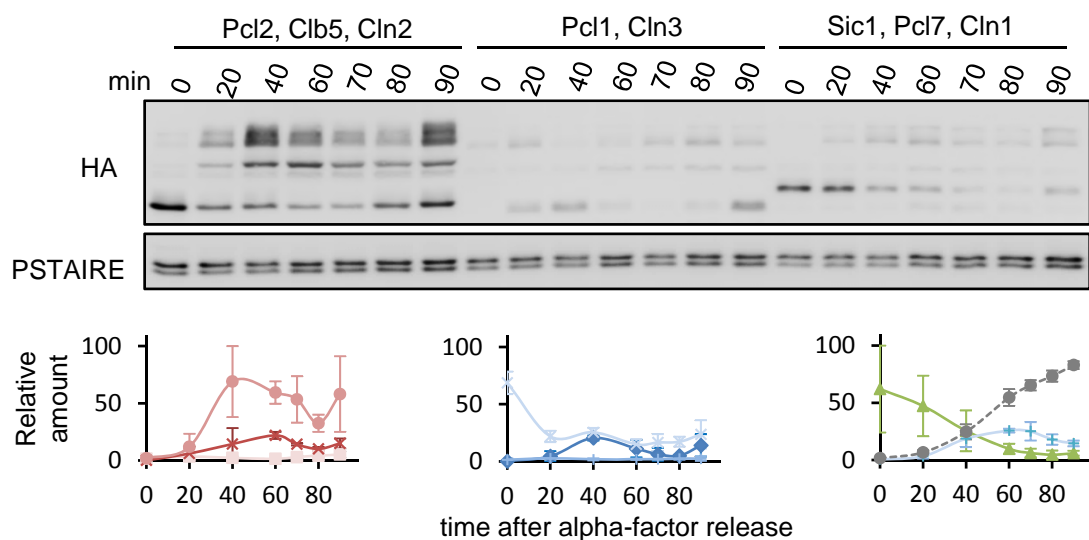

### Reductive stress

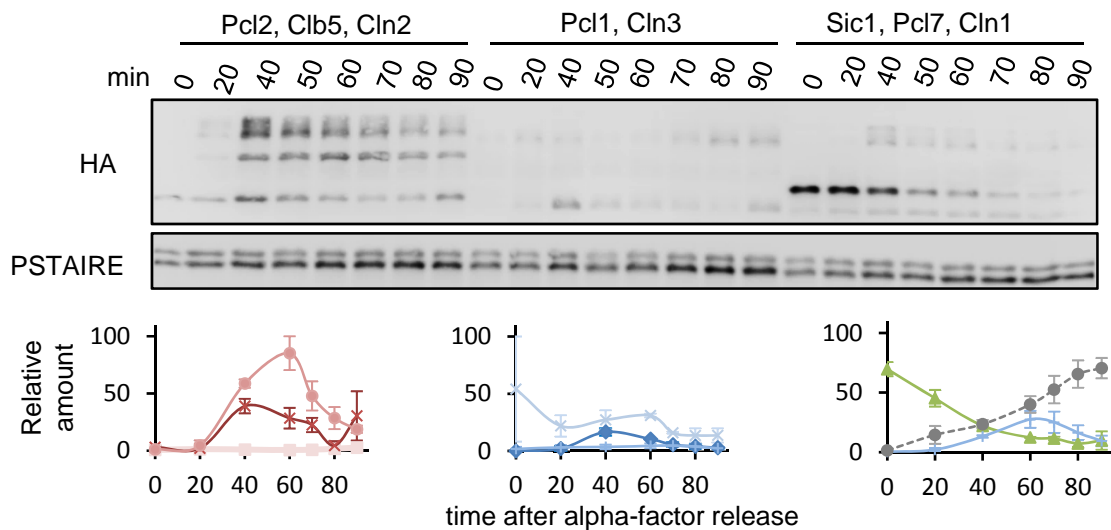

Supplement: S4 Fig — Same procedure as for S2 Fig. Cells were subjected to different stresses on α-factor release: osmotic stress (0.4 M NaCl), reductive stress (100 mM N-acetyl cysteine), and oxidative stress (10 μM menadione). A representative western blot is depicted. The graphs show mean±SEM values for at least three independent experiments. (PDF) [file pone.0218531.s004.pdf]

Fig. S5

YPD to SD, 30°C

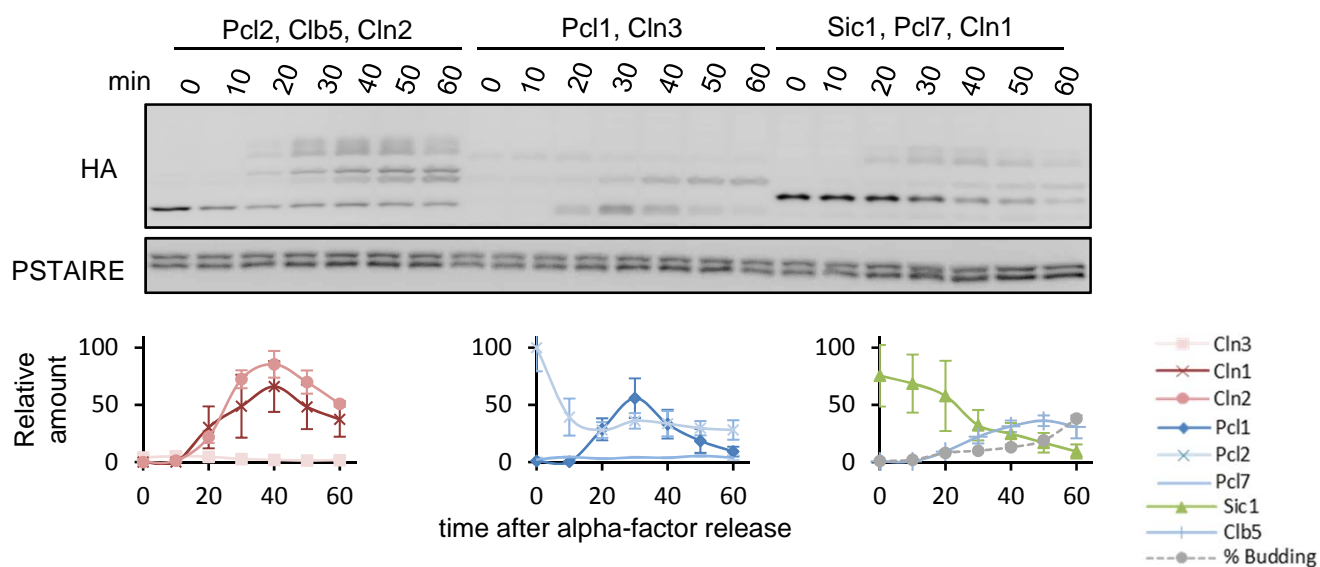

Supplement: S5 Fig — Same procedure as for S2 Fig. Cells were grown in YPD and released from α-factor arrest into an SD medium. A representative western blot is depicted. The graphs show mean±SEM values quantifying at least three independent experiments. (PDF) [file pone.0218531.s005.pdf]

Fig. S6

**Elutriation, YPD, 30°C**

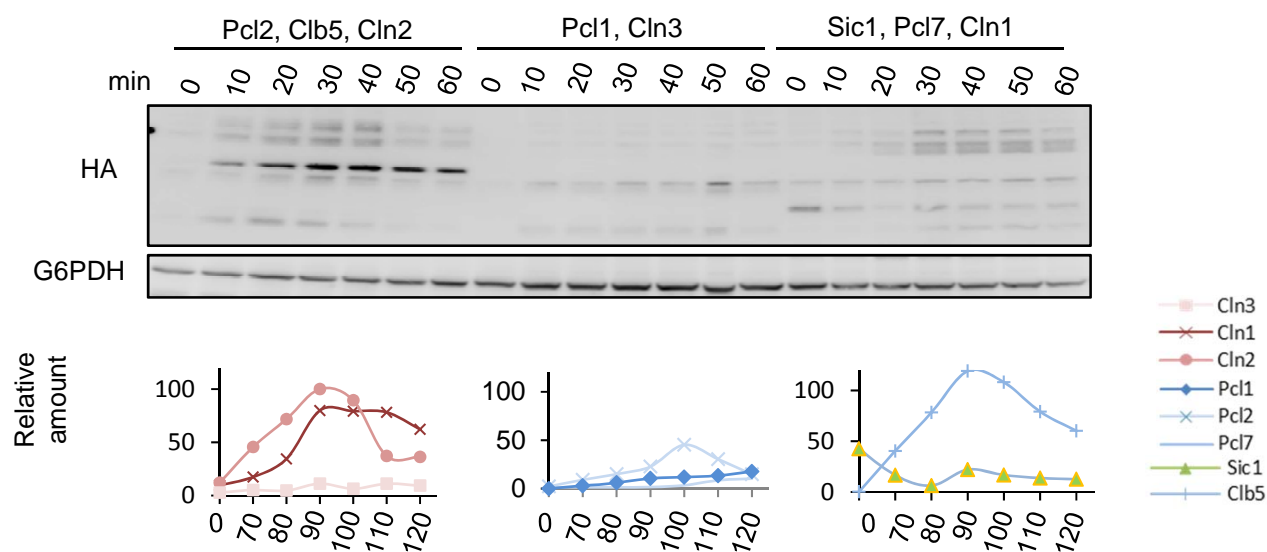

**Elutriation, YPD, heat shock**

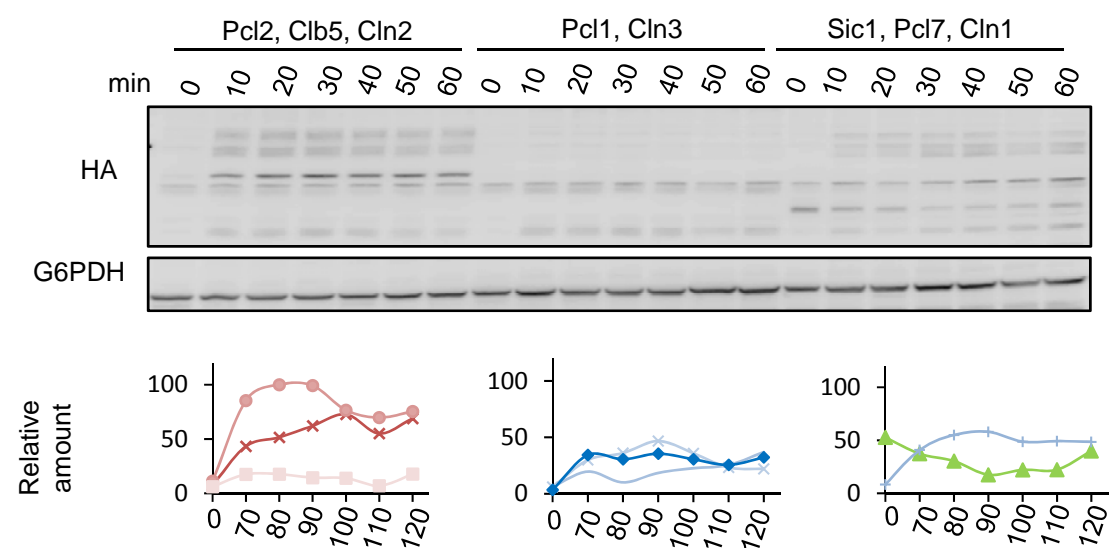

Supplement: S6 Fig — The noted strains were grown as described in methods section. Cells were synchronized by centrifugal elutriation. Time 0 corresponds to the moment of obtaining the cells form the elutriation device. After this moment, cells were incubated under agitation at 30°C (upper panel) or 37°C (lower panel). We took aliquots at the indicated times and processed them for western blot analysis as in the rest of the α-factor experiments. (PDF) [file pone.0218531.s006.pdf]
